# Simultaneous production of isopropanol, butanol, ethanol and 2,3-butanediol by *Clostridium acetobutylicum* ATCC 824 engineered strains

**DOI:** 10.1186/2191-0855-2-45

**Published:** 2012-08-21

**Authors:** Florent Collas, Wouter Kuit, Benjamin Clément, Rémy Marchal, Ana M López-Contreras, Frederic Monot

**Affiliations:** 1 Food and Biobased Research, Wageningen University and Research Centre, Bornse Weilanden 9, 6708, WG Wageningen, Netherlands; 2 IFP Energie Nouvelles, Biotechnology Department, 1-4 Avenue Bois-Préau, 92852, Rueil-Malmaison, France; 3 Current address: Clostridia Research Group, BBSRC Sustainable Bioenergy Centre, School of Molecular Medical Sciences, Centre for Biomolecular Sciences, University of Nottingham, University Park, Nottingham, NG7 2RD, UK

**Keywords:** *Clostridium*, Butanol, Biobutanol, Isopropanol, IBE fermentation, 2,3-butanediol, Acyl-CoA transferase

## Abstract

Isopropanol represents a widely-used commercial alcohol which is currently produced from petroleum. In nature, isopropanol is excreted by some strains of *Clostridium beijerinckii*, simultaneously with butanol and ethanol during the isopropanol butanol ethanol (IBE) fermentation. In order to increase isopropanol production, the gene encoding the secondary-alcohol dehydrogenase enzyme from *C. beijerinckii* NRRL B593 (*adh*) which catalyzes the reduction of acetone to isopropanol, was cloned into the acetone, butanol and ethanol (ABE)-producing strain *C. acetobutylicum* ATCC 824. The transformants showed high capacity for conversion of acetone into isopropanol (> 95%). To increase isopropanol production levels in ATCC 824, polycistronic transcription units containing, in addition to the *adh* gene, homologous genes of the acetoacetate decarboxylase (*adc*), and/or the acetoacetyl-CoA:acetate/butyrate:CoA transferase subunits A and B (*ctfA and ctfB*) were constructed and introduced into the wild-type strain. Combined overexpression of the *ctfA* and *ctfB* genes resulted in enhanced solvent production. In non-pH-controlled batch cultures, the total solvents excreted by the transformant overexpressing the *adh*, *ctfA*, *ctfB* and *adc* genes were 24.4 g/L IBE (including 8.8 g/L isopropanol), while the control strain harbouring an empty plasmid produced only 20.2 g/L ABE (including 7.6 g/L acetone). The overexpression of the *adc* gene had limited effect on IBE production. Interestingly, all transformants with the *adh* gene converted acetoin (a minor fermentation product) into 2,3-butanediol, highlighting the wide metabolic versatility of solvent-producing *Clostridia*.

## Introduction

The limited supply and the negative environmental effects of the use of petroleum-derived fuels and chemicals have stimulated efforts for the development of more environmentally-friendly processes. In this respect, the fermentation of carbohydrates into acetone, butanol and ethanol (ABE) or isopropanol, butanol and ethanol (IBE) is a promising way for the production of green chemicals and fuels. In the past, both ABE and IBE fermentations were performed worldwide at industrial scale until they were replaced by petrochemical processes (Jones and Woods 1986; Rogers et al. 2006). Many resources are currently being devoted to develop economically-viable fermentation processes based primarly on lignocellulosic biomass hydrolysates as substrates (Dürre 2007,2008; López-Contreras et al. 2010; Green 2011).

For fuel applications, the IBE mixture appears to be more attractive than the ABE one. Isopropanol shows a higher energy density than acetone (23.9 MJ/L vs 22.6 MJ/L) and this mixture has already been used as an additive for gasoline or diesel oil (Peralta-Yahya and Keasling 2010). Isopropanol can be catalytically condensed into di-isopropyl ether (DIPE) (Logsdon and Loke 2000). DIPE displays good fuel properties and could substitute methyl *tert*-butyl ether (MTBE) as isooctane index enhancer in gasoline composition (Huang and Sorensen 1990). Another important potential application of biologically-produced isopropanol is as a precursor for green propylene, which is the second most important chemical intermediate in the petrochemical industry after ethylene. Propylene is used in many chemical reactions for the synthesis of a wide variety of products, including plastic materials.

The clostridial species that produce neutral solvents (ABE or IBE) are strictly anaerobes, rod-shaped and spore-forming bacteria. Most of them, such as *C. acetobutylicum* ATCC 824, produce ABE but some others, such as *C. beijerinckii* NRRL B593, excrete IBE (George et al. 1983; Chen and Hiu 1986). ABE and IBE batch fermentations are similar, displaying a biphasic kinetic pattern (Jones and Woods 1986; Girbal and Soucaille 1998). After production of acetic and butyric acids in exponential growth, fermentation switches to formation of neutral solvents shortly before entering stationary phase. In the IBE fermentation, depending on the strain and the cultivation conditions, residual acetone may also be an end-product (Ismaiel et al. 1993).

In *C. beijerinckii* NRRL B593, the reduction of acetone into isopropanol is catalyzed by a NADPH-dependent secondary-alcohol dehydrogenase (s-Adh), which has been extensively characterized (Yan et al. 1988; Ismaiel et al. 1993; Korkhin et al. 1998; Goihberg et al. 2010). Although the s-Adh was clearly distinct from clostridial primary-alcohol dehydrogenases (Chen 1995) that reduce butyraldehyde into butanol, the s-Adh showed activity on both primary and secondary alcohols, with a preference for secondary ones (Ismaiel et al. 1993). Kinetic studies confirmed that the physiological substrate was acetone.

Metabolic engineering has been used to create pathways for isopropanol production in *Escherichia coli*. Introduction of four genes from *C. acetobutylicum* (*ctfA*, *ctfB*, *adc* and thiolase (*thl*)) into *E. coli* generated a strain capable of producing acetone (Bermejo et al. 1998). By introduction of the *C. beijerinckii adh* gene in combination with the aforementioned genes, isopropanol excretion by *E. coli* was achieved up to the concentrations of 4.9 g/L (Hanai et al. 2007) and 13 g/L (Jojima et al. 2008). The engineered *E. coli* strains surpassed the best reported wild-type clostridial strains, *C. beijerinckii* and *C*. *isopropylicum*, excreting approximately 4 g/L isopropanol (Groot and Luyben 1986; Matsumura et al. 1992). A major advantage of the engineered *E. coli* strains was the lack of important competing pathways for by-products. Recently, the *adh* gene from *C. beijerinckii* was cloned into the ABE-producing strain *C. acetobutylicum* ATCC 824. The resulting transformants excreted 6.1 g/L isopropanol and a minor amount of acetone (Lee et al. 2012).

In the present study, different IBE-producing transformants of *C. acetobutylicum* that showed high isopropanol excretion capacities have been constructed. The fermentation performances of the transformants were characterized in batch cultures using laboratory-scale bioreactors with or without pH-control and compared to those of the wild-type IBE or ABE- producing strains. In addition, formation of 2,3-butanediol by *C. acetobutylicum* transformant strains harbouring the *adh* gene was described and characterized for the first time.

## Materials and methods

### Strains and cultivation conditions

The microorganisms used are listed in Table 1. *E. coli* culture stocks were stored at −80°C in 20% (v/v) glycerol. *E. coli* strains were cultivated at 37°C with agitation (250 rpm) in LB (lysogeny broth) medium (Bertani 2004). For cultivation of *E. coli* BW25113 harbouring pMTL500E based plasmids, the LB medium was supplemented with 2% (w/w) glucose.

**Table 1:** Strains and plasmids used in this study

**Strain or plasmid**	**Relevant properties**	**References**
*E. coli* XL1 blue	*Cloning and plasmid maintenance*	
*E. coli* DH10B(pAN2)	*Plasmid methylation*	
*E. coli* BW 25113	*Ipa operons expression*	
*C. beijerinckii* NRRL B593	*Wild type, Isopropanol natural producer*	*(Georgeet al. *1983*, Chenet al. *1986*)*
*C. acetobutylicum* ATCC824		*(Jones and Woods*^1986^*)*
pMTL500E	*Empty vector (ermB*^*R*^*/amp*^*R*^)	*(Oultram et al.*^1988^*)*
-pFC002	*thlp_[adh]*	*this article*
-pFC005	*thlp_[adh; adc]*	*this article*
-pFC006	*thlp_[adh; ctfA; ctfB]*	*this article*
-pFC007	*thlp_[adh; adc; ctfA; ctfB]*	*this article*
pTHL	*thlp_*[*thl*], *kan*^*R*^	this article
pAN2	*ttc*^*R*^	(Mermelstein and Papoutsakis 1993, Heap *et al. *2007)

*Clostridium/E. coli* selective marker are indicated in parentheses.

Clostridial wild-type and transformants were stored as spore suspensions at −20°C in 15% (v/v) glycerol. Prior to the inoculation of pre-cultures, each spore suspension (500 μL) was heat-shocked in a water bath for 10 min at 70°C (*C. acetobutylicum* ATCC 824 and its transformants) or 1 min at 100°C (*C. beijerinckii* NRRL B593). Culture media for *Clostridia* were made anaerobic by sparging with nitrogen gas. Cultures and pre-cultures were performed in CM1 medium (Kuit et al. 2012) which contains, per liter: yeast extract, 5.0 g; KH_2_PO_4_, 1.0 g; K_2_HPO_4_, 0.76 g; ammonium acetate, 3.0 g; *p*-aminobenzoic acid, 0.10 g; MgSO_4_·7 H_2_O, 1.0 g; and FeSO_4_·7 H_2_O, 0.5 g, glucose, 90 g. Pre-cultures of *C. beijerinckii* were grown in medium containing 60 g/L glucose.

Batch fermentations were carried out anaerobically in 2-L (1-L working volume) Applikon glass bioreactors (Applikon, The Netherlands) using CM1 medium. When needed, pH was maintained at 5.0 by automatic addition of 4 M KOH solution. Static flask fermentations were carried out anaerobically in 120 mL serum bottles with 50 mL of CM1 medium.

For the preparation of clostridial competent cells, cells were grown on CG medium (Roos et al. 1985), as described previously (Oultram et al. 1988). When required, culture media were supplemented with ampicillin (100 μg/mL), chloramphenicol (30 μg/mL), erythromycin (50 μg/mL or 30 μg/mL for liquid cultures and plates, respectively).

Microbial growth was monitored by optical density measurements at 600 nm (Pharmacia Biotech Ultrospec 2000).

### Plasmid construction and transformation

Genomic DNA from *Clostridium* strains was isolated using GenElute bacterial genomic DNA kit from Sigma-Aldrich. Plasmid DNA from *E. coli* strains was extracted using the GeneJet plasmid miniprep kit from Fermentas. PCR amplifications were done using high fidelity PCR master mix (Roche). DNA restrictions and ligations were performed using New England Biolabs restriction enzymes (*Apa*I, *Fnu*4H1*, Spe*I, *Sph*I, *Xba*I and *Xho*I) and T4 DNA-Ligase enzyme, respectively. The oligonucleotides used are listed in Table 2 and were synthesized by Eurogenetec. Chemically competent *E. coli* strains were prepared using the Z-competent kit from Zymo-research. Kits were used according to supplier protocols.

**Table 2:** Oligonucleotides used for the plasmid constructions

**Primers**	** Sequence (5’-3’)**
thlp-for	GCATGCGAATTTAGAATGAAGTTTCTTATGCA
thlp_rev	*AAAA*GGGCCCCCATAGTTTATCCCTAATTTATACG
thl_g__rev	CTCTGTAGAACTAGTTTATAATTCTACAGAGTTATTTTTAAC
s-adh-for	*TTTT*GGGCCCTTAGACTATTAAAGGAATATTTTTAAGG
s-adh-rev	*TTTTCTCGAG*GTATAATCCTCCATGATCTATTATG
ctfAB for	CAACTAC*TCGAGA*TAATTTT*TCT*A*G*AGAATTTAAAAG**GAGG**GATTAAAATG
ctfAB rev	AATGGTA*CTAG*TTATTTTTT*GTCG*A*C*TGTTTCATAGTATTTC TTTCTAAACAGCC
adc for	AAACAA*CTC*G*A*GTTATAAT*C*TA*G*ATATAAATAAATAGGA CTA**GAGG**CG
adc rev	AAAAATA*CT*AGTTACCATTTAA*GTC*GACTCTTATTTTTATTA CTTAAG

Bases not complementary with genomic DNA are in italics;

Added restriction sites (*ApaI, SalI, speI, SphI, XbaI and XhoI,)* are underlined;

Ribosome binding sites are in bold.

The constructs used for the *C. acetobutylicum* transformation were based on the *E. coli*/*Clostridium* shuttle vector pMTL500E (Table 1). The thiolase promoter was PCR-amplified from genomic-DNA of *C. acetobutylicum* ATCC 824 using *thlp_for* and *thlp_rev* oligonucleotides and digested by *Apa*I and *Sph*I. The *adh* gene was PCR-amplified from *C. beijerinckii* NRRL B593 genomic-DNA using *adh_for* and *adh_rev* oligonucleotides and digested by *Apa*I and *Xho*I. The *thl* promoter and *adh* gene sequences were simultaneously cloned into pMTL500E plasmid (digested by *Sph*I and *Xho*I) to yield pFC002 construct. Genes from *C. acetobutylicum* ATCC 824 were amplified by PCR on genomic DNA using *adc_for* and *adc_rev* oligonucleotides for *adc, ctfAB_for* and *ctfAB_rev* oligonucleotides for *ctfA* and *ctfB*. PCR amplifications of *adc* gene and *ctfA_ctfB* genes were digested by *Xho*I and *Spe*I restriction enzymes and cloned into pFC002 digested *Xho*I and *Xba*I to yield pFC005 and pFC006, respectively. PCR amplification of *adc* gene was subsequently cloned into pFC006 digested *Xho*I and *Xba*I to yield pFC007.

Plasmid DNA constructs were introduced into chemically competent *E. coli* DH10B harbouring pAN2 (Oultram et al. 2007) for methylation prior to transformation into *C. acetobutylicum* as described earlier (Mermelstein and Papoutsakis 1993). Correct methylation was checked by restriction analysis with *Fnu*4HI. Methylated pFC002, pFC005, pFC006, pFC007 and methylated pMTL500E plasmids (Table 1) were electroporated into *C. acetobutylicum* ATCC 824 as described by Oultram et al (Oultram et al. 1988). Erythromycin-resistant colonies were cultivated in CGM liquid medium and total DNA was extracted as described above. The presence of the respective construct in the transformants obtained was confirmed by PCR on DNA extracted from the different colonies using specific oligonucleotides for the specific inserts. Transformant strains harbouring the right construct were found for all constructs (results not shown). Transformant strains were stored as spore suspensions and kept at −20°C.

The thiolase transcription unit was amplified from *C. acetobutylicum* genomic DNA using oligonucleotides *thlp_for* and *thlp_rev* (Table 2). The amplificate was cloned into pCR blunt II Topo (Invitrogen) to yield pTHL. The plasmid pTHL was cotransformed with pFC002, pFC005, pFC006, pFC007 or pMTL500E in chimiocompetent *E. coli* BW25113.

### Reduction of ketones by cell-free extracts

For preparation of cell-free extracts (CFEs) for enzymatic assays, *C. acetobutylicum* transformants and *C. beijerinckii* wild type strains were grown anaerobically in 100 mL of CM1 medium with 60 g/L glucose. After 15–20 h of culture, cells were harvested at 4°C by centrifugation at 15,000 g for 7 min (OD_600_: 1.5-2.0). Pellets were suspended in 20 mL of 50 mM sodium-HEPES buffer (pH 8.5) containing DTT (0.2 mM) and a set of protease inhibitors (Complete; Mini, Roche, 1 tablet in 50 mL) and washed twice. Pellets were then suspended in 3 mL of 50 mM sodium-HEPES buffer. Cell suspensions were frozen in liquid nitrogen and stored overnight at −80°C in anaerobic conditions. Cell suspensions were then slowly thawed, loaded in a French Press (Thermo Electron Corporation) and homogenized by two passes at 16,000 psi. When used, CFEs were kept on ice. Protein content in the CFEs was determined by the Bradford method (Biorad) with BSA as standard.

Reduction of acetone or racemic acetoin (D/L 3-hydroxy-2-butanone, Fluka) by s-Adh was carried out at 37°C in 50 mM of Tris buffer (pH 7.5) with 0.2 mM of NADPH and 50 mM of substrate. NADPH decrease was monitored by absorbance decrease at 340 nm using a Safire spectrophotometer (Tecan).

### Analytical procedures

Samples taken during fermentation were centrifuged at 20,000 g for 5 min and supernatants were stored at −20°C. Metabolite concentrations (sugars, organic acids, solvents and 2,3-butanediol) were determined by HPLC as previously described (Gosselink et al. 1995; Siemerink et al. 2011). A solution of 4-methyl valeric acid (Sigma-Aldrich) at 30 mM was used as an internal standard.

## Results

### Construction of expression vectors

The well-studied ABE-producing strain *C. acetobutylicum* ATCC 824 was engineered to be an IBE producer. For this purpose, the coding sequence of the *adh* gene from *C. beijerinckii* NRRL B593 was cloned downstream of the promoter sequence of the *thiolase* gene (*thl*) from *C. acetobutylicum* ATCC 824 to form pFC002 plasmid (Table 1). The promoter sequence of the thiolase gene was chosen in order to maximize expression of the *adh* gene, since the thiolase gene of *C. acetobutylicum* was reported to be constitutively expressed (Hartmanis and Gatenbeck 1984; Tummala et al. 1999; Alsaker and Papoutsakis 2005). To up-regulate the acetone pathway in the host organism, genes encoding the enzymes active in acetoacetyl-CoA to acetone conversion *i.e*. acetoacetate decarboxylase (*adc*) and acetoacetyl-CoA : acetate/butyrate:CoA transferase subunits A and B (*ctfA* and *ctfB*) were cloned into pFC002, downstream of the *adh* gene, resulting in the construct pFC007 (Table 1). Genes *adc*, *ctfA* and *ctfB* were expressed under the control of the *thl*-promoter. The role of each gene over expressed in pFC007 was subsequently assessed by constructing different combinations of *adh*, *adc*, *ctfA* and *ctfB* genes. The plasmid pFC005 contained *adh* and *adc* genes and pFC006 contained *adh*, *ctfA* and *ctfB* genes (Table 1).

### Expression of isopropanol pathway genes in *E. coli*

**Figure 1:**
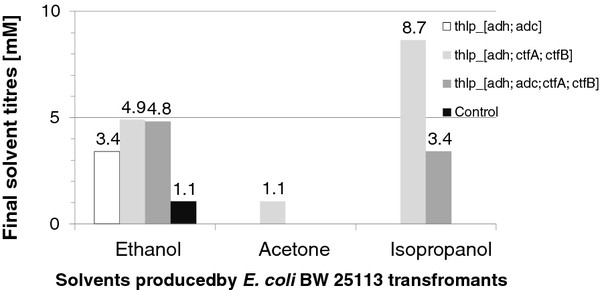
**Concentrations of ethanol, acetone and isopropanol in 48-h cultures of *E. coli *BW25113 transformants.** Each strain harboured the pTHL plasmid containing the *thl* transcription unit.

To assess their ability to promote isopropanol production, the constructs pFC002, pFC005*,* pFC006, pFC007 and pMTL500E were co-transformed with pTHL plasmid into *E. coli* BW25113. The pTHL plasmid contained the thiolase gene (*thl*) from *C. acetobutylicum* ATCC 824 (Stim-Herndon et al. 1995) to allow sufficient formation of acetoacetyl-CoA by *E. coli*. Productions of solvents by *E. coli* transformants are shown in Figure 1. All transformants produced ethanol, but only the transformants which, in addition to thiolase and *adh* genes, expressed *ctfA* and *ctfB* genes, *i.e.* transformants harbouring pFC006 (*thl*_*p*_*_adh; ctfA; ctfB*) or pFC007 (*thl*_*p*_*_adh; adc; ctfA; ctfB*) plasmids*,* produced isopropanol (3–8 mM). The *E. coli* transformants harbouring pTHL and pFC005 (*thl*_*p*_*_adh; adc*) plasmids or harbouring pTHL and pMTL500E (control) plasmids did not produce isopropanol.

### Effect of expression of the *adh* gene on the product pool of *C. acetobutylicum*

Plasmids pMTL500E, pFC002, pFC005, pFC006 and pFC007 were independently electroporated into *C. acetobutylicum*. The fermentation performance of the different *C. acetobutylicum* transformant strains was studied and compared with those of the wild-type strains (WT) in batch cultures performed in bioreactors with a 1 L-working volume. Cultures were performed with or without pH regulation. When pH regulated, the system was setup in such a way that once the pH had dropped to 5.0 it was kept at that level by the addition of KOH. Table 3 shows final fermentation performances of *C. acetobutylicum* transformants with pH regulation at 5.0.

**Table 3:** **Performance of *C. acetobutylicum *** **ATCC 824 and its transformants in 45‐h cultures performed with pH regulation at 5.0**

**Performances**	***C. beijerinckii*** **2 NRRL B593**^**2**^	***C. acetobutylicum*** **ATCC 824**					
		**WT**	**(pMTL500E)**	**(pFC002)**	**(pFC005)**	**(pFC006)**	**(pFC007)**
**Glucose consumed [g/L]**	36.8 (1.7)	62.3 (0.4)	62.5 (2.4)	53.0 (7.9)	56.3 (2.2)	68.0 (4.3)	68.1 (0.1)
**Acetic acid**^**1**^**[g/L]**	−1.7 (0.0)	0.3 (0.4)	0.4 (1.8)	1.8 (1.2)	1.2 (0.2)	1.0 (1.0)	0.0 (0.1)
**Butyric acid [g/L]**	0.3 (0.1)	1.7 (0.4)	1.3 (0.4)	2.6 (0.8)	2.5 (0.5)	2.1(1.6)	1.4 (0.0)
**Acetoin [g/L]**	0.0 (0.0)	1.1 (0.1)	0.8 (0.1)	0.0 (0.0)	0.1 (0.0)	0.0 (0.0)	0.1 (0.0)
**2,3-Butanediol (DorL) [g/L]**	0.0 (0.0)	0.0 (0.0)	0.0 (0.0)	0.5 (0.3)	0.5 (0.1)	0.6 (0.3)	0.6 (0.1)
**Ethanol [g/L]**	0.1 (0.0)	1.1 (0.2)	0.8 (0.2)	1.1 (0.3)	0.7 (0.0)	1.0 (0.3)	0.8 (0.7)
**Acetone [g/L]**	0.2 (0.0)	5.7 (0.0)	5.4 (1.0)	0.1 (0.1)	0.1 (0.0)	0.3 (0.2)	0.9 (0.9)
**Isopropanol [g/L]**	4.5 (0.3)	0.1 (0.1)	0.1 (0.1)	4.8 (0.8)	6.1 (0.1)	7.2 (0.5)	7.3 (0.3)
**Butanol [g/L]**	8.4 (1.9)	10.0 (1.5)	10.1 (1.5)	9.1 (1.6)	9.7 (0.3)	11.3 (0.8)	11.3 (0.5)
**Final solvents [g/L]**	13.2 (2.3)	16.9 (1.3)	16.4 (2.7)	15.1 (2.0)	16.7 (0.4)	19.8 (1.5)	20.4 (0.1)
**Final solvent yield [g**_**A/IBE**_**/g**_**glc.**_**]**	0.36 (0.1)	0.27 (0.02)	0.29 (0.07)	0.29 (0.04)	0.30 (0.01)	0.29 (0.02)	0.30 (0.00)
**Productivity after 30 h [g/L h]**	0.37 (0.03)	0.38 (0.05)	0.34 (0.11)	0.26 (0.13)	0.31 (0.02)	0.54 (0.11)	0.67 (0.02)
**Carbone recovery [%]**	98%	90%	86%	101%	102%	96%	95%

Cultures were performed in 1 L-working volume of CM1 containing 3.0 g/L ammonium acetate and 90 g/L glucose.

All data are given as the mean of two or three fermentations the standard error to the mean is indicated in brackets.

^1^ a negative value (−) indicates that initial acetic acid from culture medium was partially consumed.

^2^ Fermentations were carried out at pH 5.0 but the pH set-point was not reached.

Expression of *adh* by strains ATCC 824(pFC002), ATCC 824(pFC005), ATCC 824(pFC006) and ATCC 824(pFC007) resulted in the reduction to isopropanol of about 95% of the acetone natively produced. In contrast to ATCC 824 WT or ATCC 824(pMTL500E) that produced between 0.5 and 1.1 g/L acetoin (3-hydroxy-2-butanone) (Jones and Woods 1986; Xiao and Xu 2007), very low concentrations of acetoin were detected in cultures of transformants expressing the *adh* gene. However, D and/or L 2,3-butanediol (2,3-BD) accumulated at 0.5-0.6 g/L when the pH was regulated at 5.0 and at 0.6-1.2 g/L when the pH was not regulated. No *meso*-2,3-BD was identified. Production of 2,3-BD was concomitant with production of IBE. It is worth noting that 2,3-BD was not detected in cultures of *C. beijerinckii* NRRL B593, probably because the organism does not produce acetoin. The enzymatic reduction of acetone and acetoin by cell-free extracts of ATCC 824(pMTL500E), ATCC 824(pFC002), ATCC 824(pFC007) and NRRL B593 WT was tested *in vitro* (Table 4). The cell-free extracts of ATCC 824(pFC002), ATCC 824(pFC007) and NRRL B593 WT displayed significantly higher reduction activities towards acetone and acetoin than those of ATCC 824(pMTL500E) used as control (Table 4).

**Table 4:** **Reduction activities of acetone and acetoin measured in cell-free extracts of *C. beijerinckii *** **NRRL B593 and *C. acetobutylicum *** **ATCC 824 transformants harbouring pMTL500E, pFC002 and pFC007**

**Strain**		**Specific activity[μmol/min mg**_**protein**_**]**		
		**acetone**	**acetoin**	Control^*^
*C. beijerinckii*	NRRL B593	0.063	0.069	0.015
*C. acetobutylicum*	ATCC 824(pMTL500E)	0.002	0.007	0.003
*C. acetobutylicum*	ATCC 824(pFC002)	0.152	0.069	0.009
*C. acetobutylicum*	ATCC 824(pFC007)	0.076	0.118	0.018

Cell biomass used for CFE was taken from 20 h cultures.

Data are given as the mean of two replicates.

^*^ In control assays, substrate was replaced by HEPES buffer.

### Early isopropanol production in static flask culture

As the constitutive promoter of the *thl* gene was used to control gene expression (Tummala et al. 1999), the isopropanol production by ATCC 824(pFC007) was expected to start concomitantly with the production of butyric acid. Product excretion in the first hours of fermentation was studied in static flask fermentations. Butyric acid was detected prior to any solvent in all cultures of ATCC 824 transformants. The transformants expressing *ctfA* and *ctfB* genes *i.e.* harbouring pFC006 and pFC007 excreted isopropanol earlier than the wild type strain or other transformants (data not shown) and prior to any other solvent.

### Kinetics of IBE production by *C. acetobutylicum* transformants

The fermentation kinetics of ATCC 824 transformants were first investigated using a pH set-point of 5.0. The fermentation profile and performances of the control strain ATCC 824(pMTL500E) were similar to those of *C. acetobutylicum* ATCC 824 WT in the first 45 h of fermentation (Table 3). The expression of only the *adh* gene in ATCC 824(pFC002) resulted in lower solvent production (15.1 g/L IBE of which 4.8 g/L isopropanol) than ATCC 824(pMTL500E) without the *adh* gene. Moreover, the productivity of ATCC 824(pFC002) at 30 h was 25% lower than that of ATCC 824(pMTL500E). In comparison to ATCC 824(pFC002), the wild-type IBE-producer *C. beijerinckii* NRRL B593 excreted less IBE (13.2 g/L of which 4.5 g/L isopropanol), but reassimilated more efficiently the acids previously excreted. Thus NRRL B593 displayed higher solvent yield (0.36 g_IBE_/g_glc_ for NRRL B593 vs 0.29-0.30 g_IBE_/g_glc_ for ATCC 824(pFC002)).

**Figure 2:**
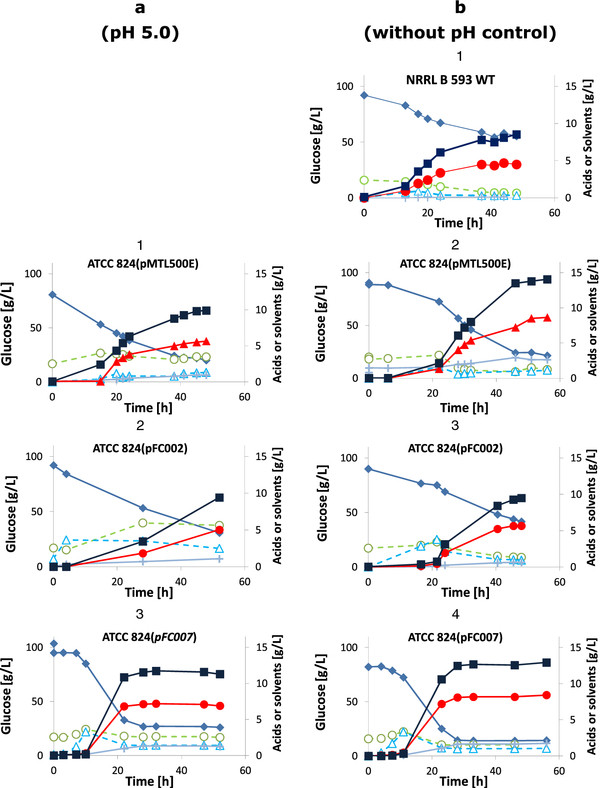
**Fermentation kinetics of *C. beijerinckii *NRRL B593 WT and *C. acetobutylicum *ATCC 824 WT and its transformants.** Column **a**: pH regulated to 5.0; column **b**: pH not regulated. Glucose (Closed diamonds), acetic acid (open circles), butyric acid (open triangles), ethanol (crosses), isopropanol (closed circles), acetone (closed triangles) and butanol (closed squares).

ATCC 824(pFC007) surpassed ATCC 824(pFC002) in the production of IBE, indicating that the overall metabolic activity was stimulated by the expression of pFC007 genes. ATCC 824(pFC007) produced more solvents (20.4 g/L IBE of which 7.3 g/L) and less acids than ATCC 824(pFC002) (15.1 g/L IBE of which 4.8 g/L isopropanol). In addition, the fermentation period was shorter and stopped about 10–15 hours earlier than for ATCC 824(pFC002) (Figure 2). Consequently, the solvent productivity after 30 h by ATCC 824(pFC007) (0.67 g/L h) was 2.6 times higher than that of ATCC 824(pFC002).

Cultures of ATCC 824(pFC005) and ATCC 824(pFC006) were performed to evaluate the contribution of each gene to the improvement of the ATCC 824(pFC007) phenotype (Table 3). Both strains produced more IBE than ATCC 824(pFC002). The combined overexpression of the *ctfA* and *ctfB* genes along with expression of *adh* conferred to ATCC 824(pFC006) a fermentation profile similar to that of ATCC 824(pFC007) (Table 3). The final concentration of acids in the ATCC 824(pFC006) culture was slightly lower than that of ATCC 824(pFC002). As with ATCC 824(pFC007), fermentations with ATCC 824(pFC006) stopped 10–15 hours earlier than with the other transformants (Figure 2). The resulting solvent productivity after 30 h (0.62 g/L h) was 2.0 times higher than ATCC 824(pFC002). In ATCC 824(pFC005), the overexpression of *adc* along with expression of *adh* gene had a more pronounced effect on the production of isopropanol (+27%) than on the production of butanol (+7%) when compared with ATCC 824(pFC002). The fermentation performances of ATCC 824(pFC005) were lower than those of ATCC 824(pFC006) or ATCC 824(pFC007) and no shortening of the fermentation period was observed. Besides, ATCC 824(pFC005), ATCC 824(pFC006) and ATCC 824(pFC007) exhibited the same solvent yield than ATCC 824(pFC002), ATCC 824(pMTL500E) and the WT.

### Effect of pH control

In order to assess the effect of the culture mode, another set of fermentations was performed without pH control (Table 5). For every strain tested, the minimum pH value reached was 4.7-4.8 (data not shown). The glucose consumptions were roughly similar to those without pH regulation. A better reassimilation of acetic and butyric acids was observed leading to better solvent titres, yields and productivities. The excretion profiles of metabolites for each strain were similar to the corresponding ones with pH regulation at 5.0. The highest solvent productions were obtained with ATCC 824(pFC007) that produced 24.4 g/L IBE (of which 8.8 g/L was isopropanol) and ATCC 824(pFC006) that produced 24.0 g/L IBE (of which 8.0 g/L was isopropanol). Lack of sporulation and extensive cell lysis were observed in cultures of ATCC 824(pFC006) or ATCC 824(pFC007) performed without pH control, highlighting the strong inhibitory effect of solvents in the early phase of cellular growth.

**Table 5:** **Performance of *C. acetobutylicum *** **ATCC 824 and its transformants in 45‐h cultures performed without pH regulation**

**Performances**	***C. acetobutylicum*** **ATCC 824**					
	**WT**	**(pMTL500E)**	**(pFC002)**	**(pFC005)**	**(pFC006)**	**(pFC007)**
**Glucose consumed [g/L]**	65.0 (1.0)	61.6 (4.2)	50.1 (5.6)	59.3 (2.5)	67.9 (7.0))	70.0 (1.9)
Acetic acid^*^[g/L]	−1.6 (0.1)	−1.8 (0.0)	−1.3 (0.1)	−2.0 (0.1)	−1.2 (0.2)	−1.4 (0.8)
**Butyric acid [g/L]**	1.1 (0.1)	1.1 (0.1)	1.0 (0.0)	1.1 (0.1)	0.6 (0.3)	0.8 (0.3)
**Acetoin [g/L]**	1.1 (0.1)	0.8 (0.0)	0.0 (0.0)	(0.0 (0.0)	0.0 (0.0)	0.1 (0.1)
**2,3-Butanediol (D** **or** **L) [g/L]**	0.0 (0.0)	0.0 (0.0)	0.6 (0.0)	0.7 (0.2)	0.8 (0.2)	1.2 (0.6)
**Ethanol [g/L]**	0.9 (0.0)	1.2 (0.4)	1.6 (0.2)	0.9 (0.1)	3.1 (1.7)	1.5 (0.6)
**Acetone [g/L]**	7.6 (0.1)	7.2 (0.0)	0.7 (0.1)	0.0 (0.0)	0.3 (0.1)	0.1 (0.1)
**Isopropanol [g/L]**	0.1 (0.0)	0.1 (0.0)	6.0 (0.5)	8.2 (0.1)	8.0 (0.4)	8.8 (0.7)
**Butanol [g/L]**	11.6 (0.2)	12.6 (1.3)	9.5 (0.3)	12.1 (0.2)	13.4 (1.5)	13.7 (1.6)
**Final solvents [g/L]**	20.2 (0.3)	21.1 (1.6)	16.2 (0.9)	21.3 (0.2)	24.0 (2.5)	24.4 (2.8)
**Final solvent yield [g**_**A/IBE**_**/ g**_**glc.**_**]**	0.31 (0.01)	0.34 (0.00)	0.33 (0.02)	0.36 (0.01)	0.35 (0.00)	0.35 (0.04)
**Productivity after 30 h [g/L h]**	0.38 (0.00)	0.42 (0.01)	0.28 (0.02)	0.30 (0.00)	0.72 (0.04)	0.80 (0.11)
**Carbone recovery [%]**	89%	94%	102%	100%	105%	101%

Cultures were performed in 1 L-working volume of CM1 containing 3.0 g/L ammonium acetate and 90 g/L glucose.

All data are given as the mean of two or three fermentations the standard error to the mean is indicated in brackets.

^*^ a negative value (−) indicates that initial acetic acid from culture medium was partially consumed.

## Discussion

In the latest developments related to the ABE fermentation process, acetone was considered to be indesirable co-product, whereas butanol is the main product of interest. Over the past few decades, various strategies have been developed to decrease the production of acetone and increase the production of butanol (Nair et al. 1994; Nair and Papoutsakis 1994; Harris et al. 2000; Sillers et al. 2008; Jiang et al. 2009; Sillers et al. 2009; Han et al. 2011). The intracellular conversion of acetone into isopropanol was an attractive alternative to avoid acetone excretion and produce a valuable alcohol. The *C. beijerinckii* NRRL B593 strain reduced acetone naturally thanks to a secondary-alcohol dehydrogenase (s-Adh) but the final titres of solvents by the NRRL B593 (George et al. 1983; Survase et al. 2011) were lower than those of the best ABE producers (Monot et al. 1982; Qureshi and Blaschek 1999).

In this study, we have constructed four plasmids harbouring the *adh* gene from *C. beijerinckii* NRRL B593 and the genes from *C. acetobutylicum* ATCC 824 that are part of the metabolic pathway from acetoacetyl-CoA to acetone. The cloned genes were successfully expressed in both *E. coli* BW25113 and *C. acetobutylicum* ATCC 824. In *E. coli*, the expression of *ctfA* and *ctfB* along with *adh* and *thl* genes allowed for the production of isopropanol. The lack of the *adc* gene did not prevent the decarboxylation of acetoacetate by *E. coli* harbouring pFC006, probably because of the instability of the molecule in acidic conditions (Hay and Bond 1967). The final concentration of isopropanol in cultures of *E. coli* strains expressing *ctfA* and *ctfB* genes (pTHL and pFC006 or pTHL and pFC007) was lower than those previously reported by other groups (Hanai et al. 2007; Atsumi and Liao 2008; Jojima et al. 2008; Yoshino et al. 2008). In our study, *E. coli* cultures were not optimised, but carried out with the purpose of checking the validity of each construct.

The plasmids were electroporated in *C. acetobutylicum* ATCC 824. The expression of *adh* gene allowed transformants to reduce natively produced acetoin and acetone to 2,3-BD and isopropanol, respectively. Either the D or L forms of 2,3-BD or a combination of both but no *meso-2,3-BD* was produced. The achiral HPLC used in the present study did not differentiate between the D and L enantiomers. Since the activity of s-Adh on acetoin had never been described, this result extends the range of substrates known for this enzyme (Ismaiel et al. 1993). Recently, the production of 2,3-BD by *C. acetobutylicum* transformants expressing an acetoin reductase (*acr*) from *C. beijerinckii* NCIMB 8052 was reported (Siemerink et al. 2011). The resulting strains also produced 2,3-BD but did not produced isopropanol. For future applications, the production of 2,3-BD by ATCC 824 transformants is still very far from that of *Klebsiella pneumoniae* (up to 150 g/L of 2,3-BD) (Ma et al. 2009).

Each transformant of ATCC 824 was characterised in a batch culture either with pH regulation at 5.0 or without pH regulation. All transformants of ATCC 824 and the wild type displayed higher solvent production levels when grown without pH-regulation. The solvent yield based on glucose consumption did not depend on the genetic modifications, but rather on the culture conditions (pH control or not). Acid assimilation was improved in the cultures without pH regulation, as also suggested by the increase of the C3 compound (acetone or isopropanol) productions. When the pH was not regulated, the pH value of the culture dropped below 5.0, increasing the concentrations of the protonated form of the acids. This has been associated with the onset of solventogenesis (Monot et al. 1984; Hüsemann and Papoutsakis 1988). Therefore, the high level of protonated acid forms in pH not-regulated cultures of ATCC 824 transformants might trigger solventogenesis at a lower concentration of total acids (protonated plus ionized) and drive more the carbon flux towards butanol or ethanol formation.

The expression of only the *adh* gene lowered total solvent production by ATCC 824(pFC002) compared to the wild type and the transformant harbouring the empty vector (pMTL500E). The lower solvent excretion by ATCC 824(pFC002) could be explained by the higher toxicity of isopropanol compared to acetone, as suggested by the octane/water partition coefficients (logK_ow_) values *i.e.* 0.05 for isopropanol and −0.25 for acetone (Yaws and Sachin 1999). The logK_ow_ was reported to be a good estimation for solvent toxicity (Vermue et al. 1993; Heipieper et al. 1994), high logK_ow_ compounds are generally more toxic than compounds with lower value. It has to be noted that the final IBE concentration of ATCC 824(pFC002) cultures (16 g/L) was still higher than that of NRRL B593 cultures (13 g/L) suggesting that the solvent sensitivity is a strain-dependent characteristic.

Under all culture conditions tested, the overexpression of all genes encoding enzymes of the acetone route (*ctfA*, *ctfB* and *adc*), along with expression of *adh* gene, conferred to ATCC 824(pFC007) high solvent production rate and high final solvent titres. The use of *thl* promoter to control the expression of *ctfA* and *ctfB* genes initiated excretion of isopropanol before those of other solvents. Recently, (Lee et al. 2012) have developed a transformant comparable with ATCC 824(pFC007) in which expression of isopropanol pathway genes were controlled by two *adc* promoters. In batch culture with pH regulation at 5.0, the maximal end-concentration of IBE was only 17.1 g/L of which 6.1 g/L was isopropanol. The difference in solvent productions observed in the two studies might result from the culture mode applied. Fed-batch with gas stripping was used and was found to improve IBE production by 35.6 g/L (Lee et al. 2012) but this type of process has never been scaled up.

The role of each gene involved in the pathway from acetoacetyl-CoA to acetone in the enhancement of ATCC 824(pFC007) fermentation performances was clarified by expressing two derivative plasmids. The overexpression of the *ctfA* and *ctfB* genes increased both the speed and the extent of acid assimilation while the overexpression of the *adc* gene had a little effect (Table 3).

This result indicates that decarboxylation of acetoacetate is not the real bottleneck. In a previous study on ABE production by ATCC 824, the overexpression of *ctfA*, *ctfB* and *adc* genes controlled by the *adc* promoter was studied at pH 5.5 (Mermelstein et al. 1993). As with our results, the combined overexpression of *ctfA, ctfB* and *adc* increased the solvent production by transformants, whereas expression of *adc* gene alone had little effect. Unlike our results, the combined expression of *ctfA* and *ctfB* genes without *adc* was found to have a limited effect. Therefore, the impact of *ctfA* and *ctfB* overexpression observed in our study might have been supported by the chemically acid-calalysed decarboxylation of acetoacetate (Hay and Bond 1967).

## Conclusion

The expression of *ctfA* and *ctfB* genes along with the *adh* gene in *C. acetobutylicum* appears to be a promising way for constructing efficient isopropanol/ethanol producers. The transformants in the present study produce the highest total IBE concentration reported for clostridial batch cultures without online IBE removal (24.4 g /L). As the IBE alcohol mix is considered to be a valuable fuel additive, the transformants obtained represent a step forward towards the development of an industrial IBE process for the production of biofuels.

## Competing interest

The authors declare that they have no competing interests.
